# Experiment of GBR for repair of peri-implant alveolar defects in beagle dogs

**DOI:** 10.1038/s41598-018-34805-w

**Published:** 2018-11-08

**Authors:** HuiPing Li, JiSi Zheng, Shanyong Zhang, Chi Yang, Yong-Dae Kwon, Yong-Jin Kim

**Affiliations:** 1 Department of Oral and Maxillofacial Surgery, Ninth People’s Hospital, Collage of Stomatology, Shanghai Jiao Tong University School of Medicine; Shanghai Key Laboratory of Stomatology & Shanghai Research Institute of Stomatology, Shanghai, People’s Republic of China; 2 Department of Oral and Maxillofacial Surgery, College of Dentistry, Kyung Hee University, Gwangneung, South Korea; 3 Department of Oral and Maxillofacial Surgery, Insan Apsun Dental Clinic, Gwangneung, South Korea

## Abstract

To guide barrier membrane choice in the treatment of peri-implant alveolar bone defects, we evaluated guided bone regeneration (GBR) using titanium (Ti) mesh or Bio-Gide membrane, independently or in combination, for repair of alveolar bone defects in Beagle dogs. Six months after extraction of the mandibular premolars and first molars from three beagle dogs, we inserted implants assigned into 3 groups and covered with the following membrane combinations: Group A: Implant + Bio-Oss + Ti-mesh, Group B: Implant + Bio-Oss + Bio-Gide, and Group C: Implant + Bio-Oss + Ti-mesh + Bio-Gide. At 6 months, micro-CT revealed that bone volume/total volume (BV/TV), trabecular number (Tb.N), and trabecular thickness (Tb.Th) was significantly greater in Group C than the other two groups, while trabecular separation (Tb.Sp) was significantly lower, suggesting improved bone regeneration. The distance between bands of three fluorescent tracking dyes was significantly greater in Group C, indicating faster deposition of new bone. The Bio-Oss particles were ideally integrated with newly deposited bone and bone thickness was significantly larger in Group C. These findings suggest that combination of Bio-Gide membrane and titanium mesh can effectively repair peri-implant alveolar bone defects, achieving enhanced bone regeneration compared to titanium mesh or Bio-Gide alone, and therefore providing a novel treatment concept for clinical implant surgery.

## Introduction

Adequate bone volume is an important prerequisite for a predictable long-term prognosis in implant dentistry. However, in some cases, due to insufficient bone at the labial and/or buccal sides of the jaw, normal anatomical structures such as the maxillary sinus and mandibular nerve canal, or other reasons, the clinical situation does not always allow an implant to be ideally placed into the alveolar bone for optimal aesthetics and functional superstructure^1–3^. Rebuilding bone where there is no supportive space and limited nourishment is difficult to achieve, and addressing alveolar defects between the implant and the extraction socket is one of the most important factors influencing the success rate of tooth implant. Therefore, various methods have been developed to increase bone volume and augmentation^4^.

GBR is a technique that uses a mechanical barrier to prevent soft tissue from growing into a bony defect before osteogenic cells can seal the gap and develop new bone matrices for osseous regeneration^5^. In many ways, GBR is reported to provide the best and most predictable results when employed to fill peri-implant bone defects with new bone. Furthermore, GBR improves the predictability of bone augmentation and provides long-term stability to the newly augmented site. The GBR therapeutic protocol involves surgical placement of a cell occlusive membrane facing the bone surface, in order to physically seal off the skeletal site in need of regeneration^6^. During the regeneration process, the membrane creates and maintains a secluded space, thus providing an environment that is permissive for recruitment and proliferation of osteoprogenitor cells, differentiation along the osteoblastic lineage, and expression of osteogenic activity. Various non-resorbable and resorbable membrane materials have been used in experimental and clinical studies in the context of GBR treatment^7–9^. Current barrier membranes should fulfill the main design criteria for GBR, such as biocompatibility, occlusivity, spaciousness, clinical manageability, and appropriate integration with the surrounding tissue. At present, the Bio-Gide membrane is widely used. It consists of collagens I and III, without other organic components. The Bio-Gide membrane can reportedly achieve perfect tissue barriers, induces the deposition of new bone, has a stable absorption rate, and does not cause inflammation, inhibit bone formation, or accelerate bone resorption^10–12^. However, the Bio-Gide membrane is too soft to endure masticatory pressure on alveolar bone, and therefore provides limited space and mechanical support to the tissue during bone formation. Considering that the alveolar bone response to pressure may cause bone resorption, the occlusal pressure on implant sites may result in implant failure.

As early as the 1990s, Simion *et al*.^13^ reported the use of titanium mesh in GBR to repair severe alveolar defects at implant sites. Recently, the use of a rigid titanium mesh, stabilized as a tent over a resorbed ridge in full/partial restoration of the alveolar process, has been reported^14,15^. The porous titanium mesh can cover the alveolar defects to form the ideal shape of the alveolar ridge, thus effectively preventing soft tissue collapse and displacement of bone particles during bone regeneration^16,17^. In addition, due to its high plasticity, titanium mesh can be bent into different shapes to fit the contour of the alveolar ridge during clinical surgery^18^. Compared to most biomembranes, titanium mesh has a low postoperative exposure rate^19^. Furthermore, even if the titanium mesh is exposed postoperatively in rare cases, there is no urgent need to remove it because its porous structure can allow vessels to enter deeper tissues without preventing blood flow^20^.

In the present study, we performed a comparative analysis of implant outcomes using the combination of Bio-Gide (Osstem, Seoul, Korea) and titanium mesh (Osstem, Seoul, Korea) as a framework for a filler containing bovine-derived xenograft (Bio-Oss Collagen, Geistlich Biomaterials, Wolhusen, Switzerland).

## Results

### Micro-CT

**Figure 1:**
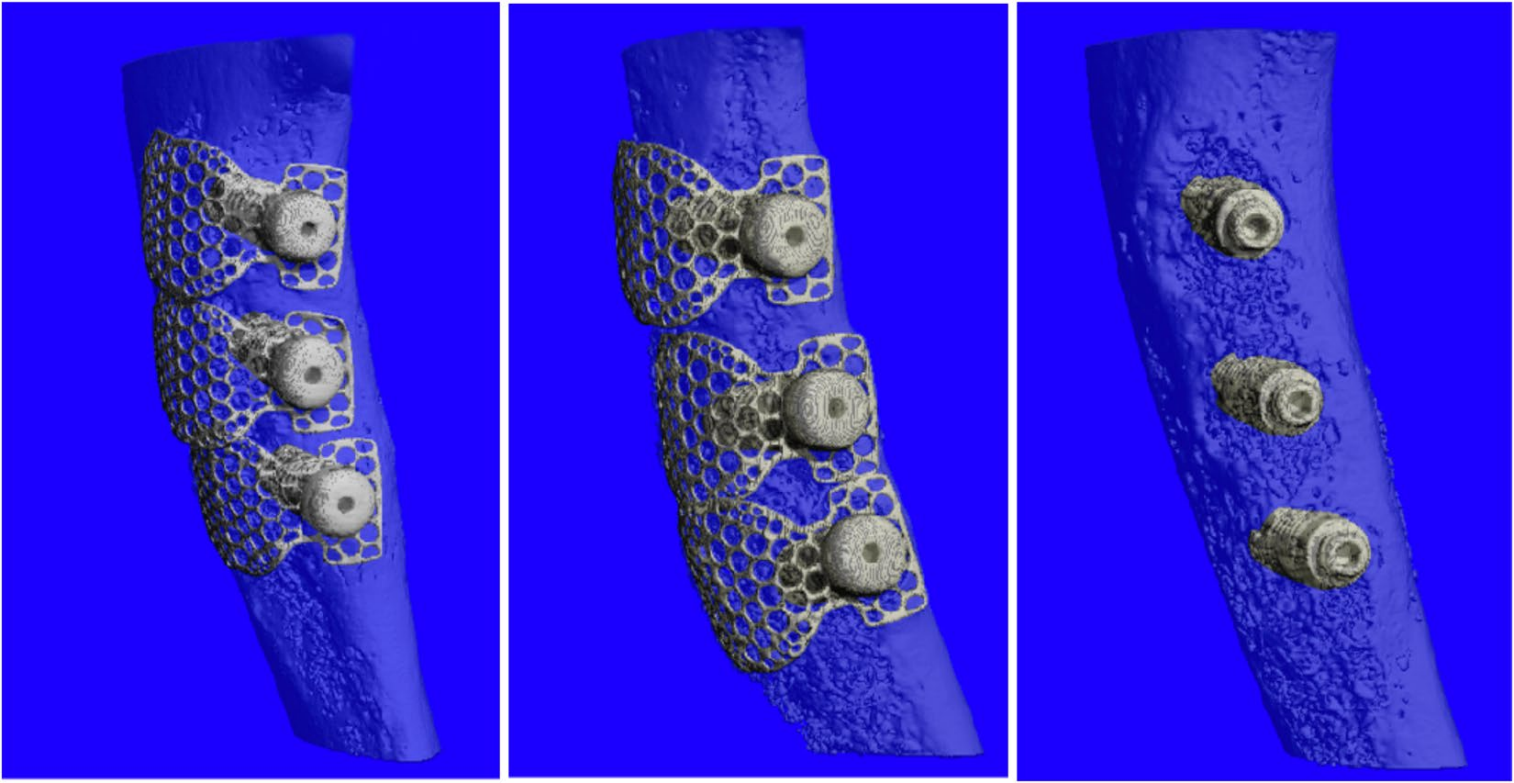
Micro-CT analysis of alveolar bone volumetric parameters. (**A**) Three-dimensional reconstruction of Group A. (**B**) Three-dimensional reconstruction of Group B. (**C**) Three-dimensional reconstruction of Group C. (**D**) Bone volume/total volume (BV/TV). (**E**) Trabecular number (Tb.N). (**F**) Trabecular separation (Tb.Sp). (**G**) Trabecular thickness (Tb.Th). (*P < 0.05; **P < 0.01).

Three-dimensional reconstruction was carried out to analyse morphological outcomes and degree of bone regeneration (Fig. 1A–C). Bone volume/total volume (BV/TV), trabecular number (Tb.N), trabecular separation (Tb.Sp), and trabecular thickness (Tb.Th) were measured from three-dimensional reconstruction images (Fig. 1D–G). Micro-CT with three-dimensional reconstruction revealed that bone regeneration was significantly enhanced in Group C compared to the other two groups (*P* < 0.01). No significant difference was observed between Group A and Group B.

### Fluorescence microscopy

**Figure 2:**
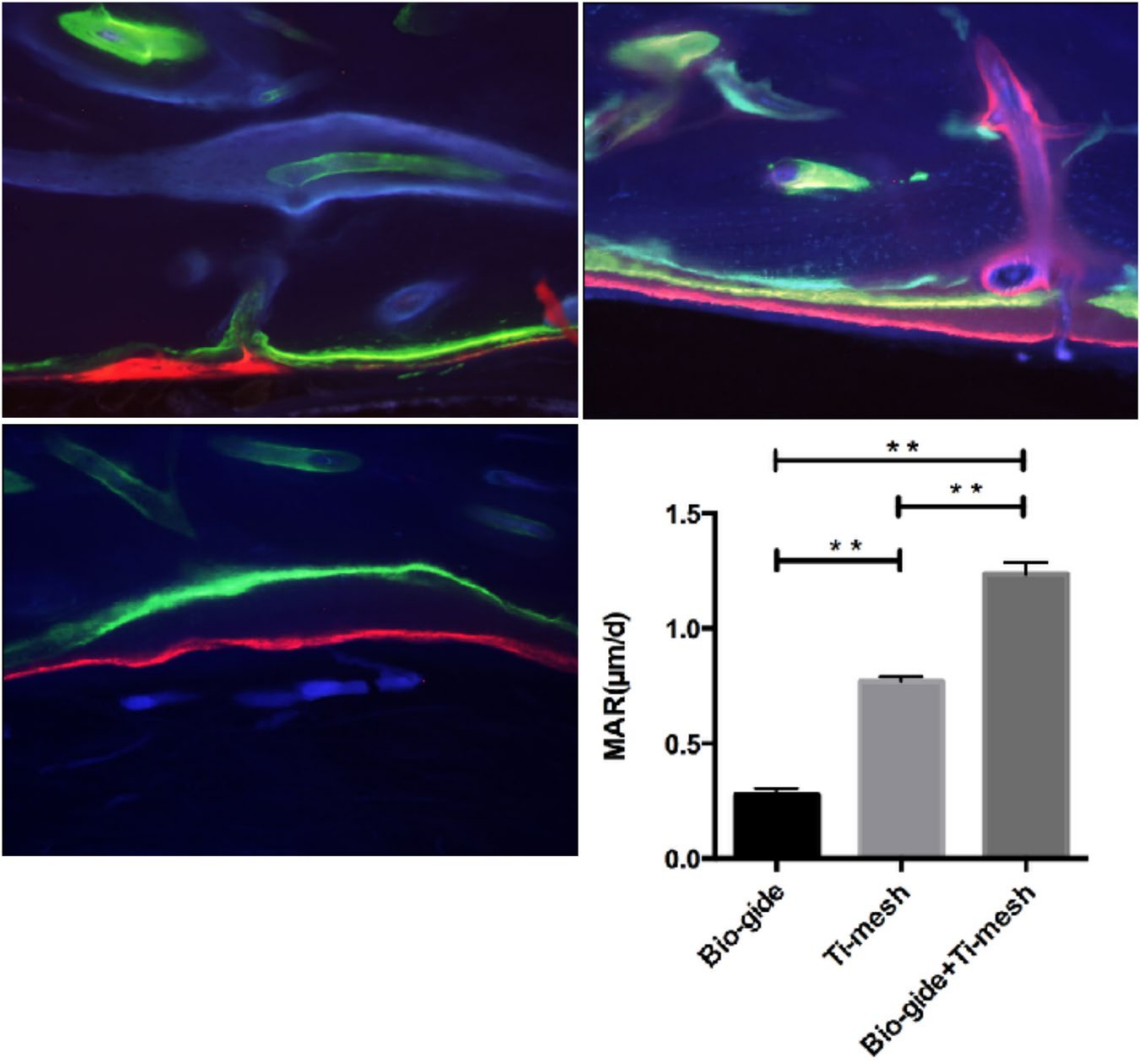
Fluorescence microscopy of newly formed bone. (**A**) Image of Group A shows the three different fluorescent dyes almost coincident, suggesting little bone deposition. (**B**) Image of Group B shows a relative further distance between dyes. (**C**) Compared with the other two groups, the distance between the bands of three fluorescent dyes was significantly greater in Group C (*P* < 0.01), indicating faster deposition of new bone.

Fluorescent sequential labeling of newly formed bone was visualized using different fluorescent dyes. The different colored fluorescent dyes enabled a description of the time course of new bone formation. Integration of the fluorescent dyes into bands of newly deposited bone was visualized by fluorescence microscopy. The distance between the bands of three fluorescent dyes was significantly greater in Group C (*P* < 0.01), indicating faster deposition of new bone (Fig. 2A–C). There was no significant difference between Group A and Group B.

### Histomorphometry

**Figure 3:**
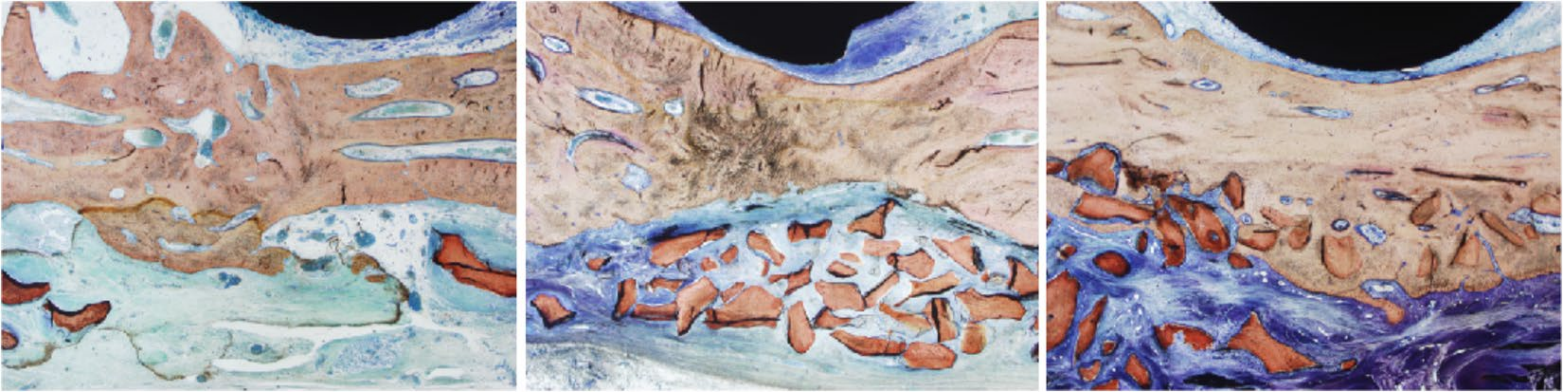
Histomorphometry of Verhoeff-Van Gieson staining shows fibroblasts (blue) and mature bone trabeculae (brown) of the defect area. (**A**) In Group A, there was massive soft tissue admixed with bone tissue, and most of the Bio-Oss particles were absent. (**B**) In Group B, there was more remaining Bio-Oss particles and less soft tissue admixed with bone tissue. (**C**) In Group C, the Bio-Oss particles were perfectly integrated with the newly deposited bone and the bone thickness was significantly larger than the other two groups (*P* < 0.01).

Verhoeff-Van Gieson staining shows fibroblasts (blue) and mature bone trabeculae (brown) within the defect area. In Group A (Fig. 3A), we observed massive soft tissue admixed with bone tissue, which may be inflammatory hyperplasia, mimicking osteoid or chondroid tissue. Most of the Bio-Oss particles were absent, and the remaining particles were surrounded by soft tissue instead of integrating with the bone. Group B (Fig. 3B) appeared similar to Group A, but there were more remaining Bio-Oss particles and less soft tissue admixed with bone tissue. In contrast to Group A and Group B, Group C (Fig. 3C) showed perfect integration of the Bio-Oss particles with the newly deposited bone, and the bone thickness was significantly larger (*P* < 0.01) than the other two groups.

## Discussion

The concept of GBR for the reconstruction of alveolar ridge defects and simultaneous implant placement was put forward to provide a sufficient volume and quality of alveolar bone at potential implant recipient sites. The basic principle of GBR involves the placement of mechanical barriers to isolate the bone defect from the surrounding connective tissue, thus providing bone-forming cells with access to a secluded space intended for bone regeneration^21^. Traditionally, alveolar ridge reconstruction was achieved with various membrane barriers to prevent soft tissue ingrowth. Popular membrane barriers include non-resorbable e-PTFE and resorbable barriers composed of collagen, polylactic acid, and polygalactin^22^.

According to previous studies, Bio-Gide membrane could provide occlusivity to tissue formation, which may prevent or delay bone formation. Occlusivity is closely linked to membrane porosity and this factor has a major influence on the potential for cell invasion^23^. Aside from its advantages, Bio-Gide membrane was too soft to bear the masticatory pressure. Considering the characteristic of alveolar bone that compression results in absorption and distraction results in osteogenesis, the masticatory compression force on alveolar bone may cause bone absorption, leading to failure of implant surgery. Since no resorbable or nonresorbable membrane can presently perfectly achieve the required alveolar height due to space restrictions, research using animal and clinical studies is still ongoing in order to establish an ideal membrane for treatment.

Titanium is a metal with excellent biocompatibility and has been used in numerous surgical applications^24,25^. As an augmentation housing device, titanium mesh has been shown to be rigid enough to prevent soft tissue collapse and functions predictably in both lateral and vertical bone augmentation^26^. The oval optimal pore size of the mesh is about Ф1.0 mm to avoid any dispersion of the grafted particle, and to increase the blood supply of the surgical site. In addition, smooth surfaced titanium barriers are less susceptible to bacterial contamination than resorbable membranes. Since every membrane offers both advantages and disadvantages, a membrane should be selected based on a thorough understanding of the benefits and limitations inherent to the materials in relation to the functional requirements in the specific clinical application. In this case, we combined the Bio-Gide membrane and titanium mesh to achieve an improved bone regeneration effect.

We used a one-stage approach, with simultaneous bone augmentation and placement of dental implants. Since the GBR and implantation procedure are performed simultaneously during the same surgical process, bone regeneration and implant osseointegration occur during the same period, and may thus result in an improved osseointegration effect and achieve a sufficient bone mass to facilitate implants. Analysis of our micro-CT data showed significantly enhanced bone formation using the combination of Bio-Gide membrane and titanium mesh. In addition, Verhoeff-Van Gieson staining showed significantly larger newly deposited bone and bone thickness compared to the other two groups. The overall histological and radiographic results show a survival and success rate of 100% according to Albrektsson *et al*.^27^, confirming our previous hypothesis that the combination of Bio-Gide membrane and titanium mesh may achieve an enhanced bone regeneration effect.

A thorough understanding of the benefits and limitations inherent to various materials in specific clinical applications will be of great value and benefit in the selection of an optimal membrane for GBR. The mechanical properties and design of the titanium mesh ensure optimal graft integration by firm immobilization and contour stability. However, necessary adjustments to the pore size and frequency in titanium mesh biomaterials should improve their efficacy in dental applications. In the present study, a 3D customized pre-formed design titanium mesh offers an excellent solution for GBR in dental applications over other membrane types. If we could apply the 3D customized pre-formed design titanium mesh and biopolymer composite membranes to GBR, the overall post-operative healing course will be excellent.

## Methods

### Ethics statement

This study was approved by the Ethics Committee of Shanghai Jiao Tong University School of Medicine. All methods were performed in accordance with the Guiding Principles for the Care and Use of Laboratory Animals of Shanghai Jiao Tong University School of Medicine. The study was conducted at the Research Institute for Animal Breeding and Nutrition, Shanghai Jiao Tong University School of Medicine. All materials were offered by Osstem, Seoul, Korea, unless stated otherwise.

### Animals and experimental design

Three male beagle dogs (mean age 18 months, mean weight 12.5 kg) were used for the study. All operations and measurements were performed under general anaesthesia using intravenous injections of ketamine hydrochloride (2.5 ml/10 kg; Ketavet 10%, Pfizer, Berlin, Germany) and xylazine hydrochloride (1 ml/10 kg; Xylavet 2%, Sanofi-Aventis, Budapest, Hungary) administered every 15 min. For pain control, Metamizole (1 ml/10 kg; Algopyrin, Sanofi-Aventis, Budapest, Hungary) was injected intramuscularly after completion of the operations and was continued for 3 days. To prevention infections, intramuscular injection of amoxicillin hydrochloride solution (150 mg/ml, 1 ml/10 kg, Pfizer, Berlin, Germany) was administered after surgery for 2 weeks.

### Creation of an alveolar bone defect model

After induction of general anaesthesia as described above, lingual and buccal mucoperiosteal flaps were elevated at both mandibular sides. The teeth from the first premolar to the first molar were separated using burs and extracted with forceps. The residual alveolar bone was ground as smoothly as possible to ensure that there was no bony spur. Excess soft tissue was removed, and flaps were closed using resorbable sutures (Vicryl 3.0, Ethicon, Norderstedt, Germany). Considering the difficulty of chewing after teeth extraction, all the animals received liquid diets for 2 weeks post-surgery. Alveolar bone was allowed to heal for 6 months before proceeding with implant surgery.

### Implant surgery procedures

**Figure 4:**
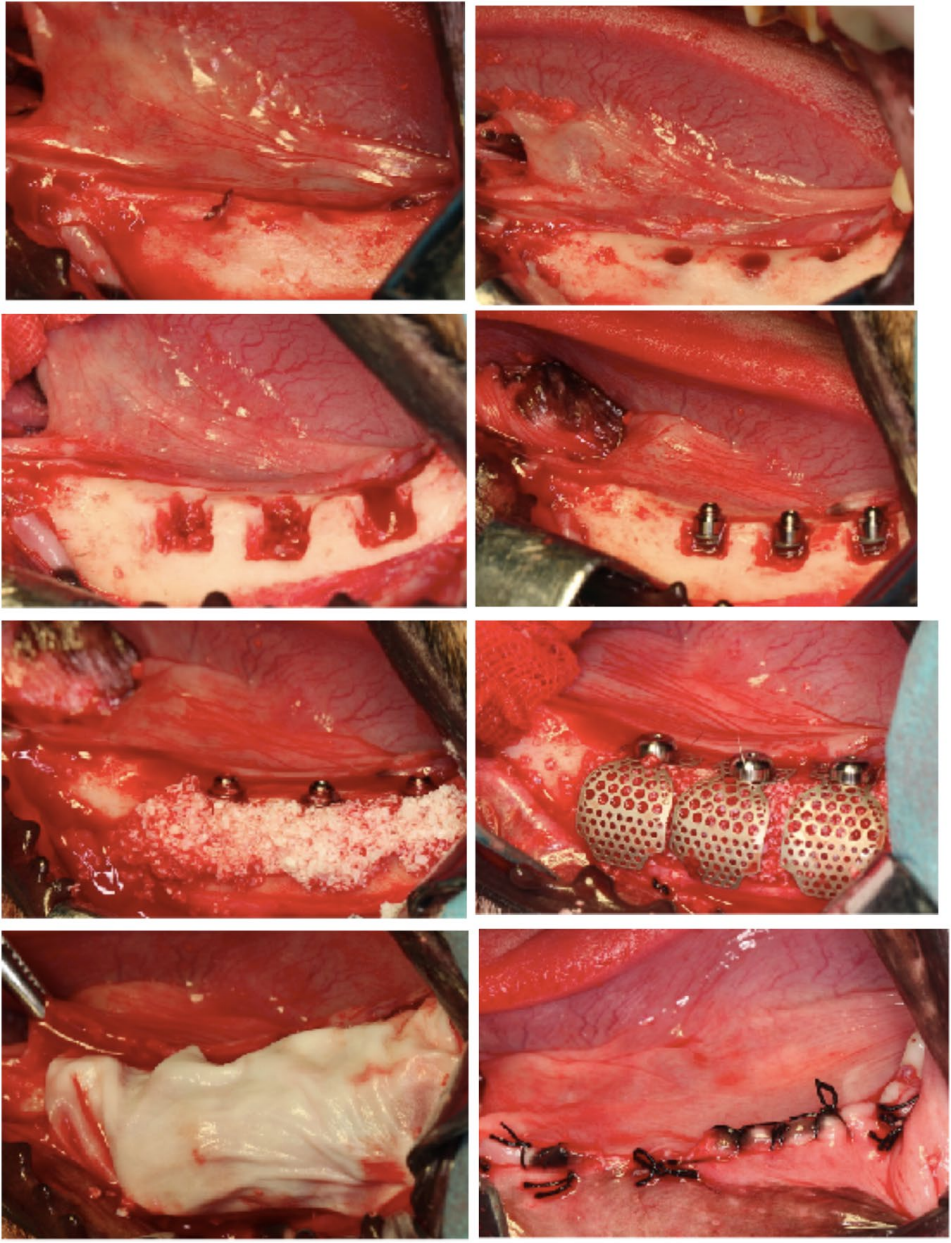
Implant surgery process. (**A**) Elevation of lingual and buccal mucoperiosteal flaps. (**B**) Preparation of implant site. (**C**) Preparation of buccal bone defect (4 mm × 4 mm × 4 mm). (**D**) Implant placement. (**E**) Bio-Oss mixed with blood was applied to fill the bone defect. (**F**) Ti-mesh, fixed by a height, was set as an augmentation housing device. (**G**) Bio-Gide absorbable membrane was placed to create a microenvironment. (**H**) The flaps were closed using absorbable sutures.

Six months after teeth extraction, implant surgery was performed. A week before implant surgery, computed tomographic (CT) scans were performed on each animal with a 64-slice spiral imager (slice thickness 0.625 mm) (Light Speed Ultra; General Electric, Millwaukee, WI). The data from the CT scans in the DICOM format were entered into an interactive Simplant software program (Version 11.04, Materialise Medical, Leuven, Belgium) to reconstruct three-dimensional surface models of each animal. From the CT scans, the height from the alveolar ridge to the mandibular canal and buccal to lingual width were measured. Preoperative design was carried out carefully to ensure the precise location of the implants. For the 3 animals, there were a total of 6 sides (3 implants/side) at the mandible, which were allocated into 3 groups: Group A: Implant + Bio-Oss + Ti-mesh, Group B: Implant + Bio-Oss + Bio-Gide, and Group C: Implant + Bio-Oss + Ti-mesh + Bio-Gide. After induction of general anesthesia, a midcrest incision was made for the elevation of lingual and buccal mucoperiosteal flaps at both mandibular sides (Fig. 4A). The implant bed was prepared according to the standard protocol (TSIII SA Implant System, Osstem, Seoul, Korea; Fig. 4B). After preparation of the implant site, the buccal bone defect was made as large as 4 mm × 4 mm × 4 mm (Fig. 4C). However, the implant (Ф3.5 mm × 8.5 mm, Osstem, Seoul, Korea) was chosen 2 mm shorter than the depth of the final drill in order to place the implant shoulder submucosally for a submerged implant and bone graft healing (Fig. 4D). Bio-Oss mixed with blood was applied to fill the bone defect. In group A, the Ti-mesh, fixed by a height, was set as an augmentation housing device. In group B, the Bio-Gide absorbable membrane was placed to create a microenvironment for bone healing and prevent the soft tissue from growing into a bony defect. In group C, the Ti-mesh and Bio-Gide were used in combination. The cover cap was then set and flaps were sutured using absorbable sutures (Fig. 4E,H). The animals were respectively injected with tetracycline hydrochloride (Sigma Chemical Co, St. Louis, MO, USA; 25 mg/kg), calcein (Sigma Chemical Co, St. Louis, MO, USA; 20 mg/kg) and alizarin red (Sigma Chemical Co, St. Louis, MO, USA; 30 mg/kg) at 1, 3, and 5 months after implant surgery. All dyes were prepared immediately before use with 2% sodium bicarbonate or saline. After preparation, pH was adjusted to 7.4 and the solution was filtered through a 0.45 mm filter (Schleider & Schuell GmbH, Dassel, Germany). Each animal received a total dose of 3 ml^28^.

### Preparation of specimens

The animals were sacrificed 6 months after implant surgery. The block sections of the mandible containing the implants and surrounding tissues were dissected free and the soft tissue was roughly removed. The bone was fixed in PBS-buffered formaldehyde (3.7%) solution (pH 7.4) for 2 days at 4 °C, washed with PBS for 2 days, and used for further studies.

### Micro-CT scanning

The fixed mandibles were analyzed using a high-resolution micro-CT (Skyscan 1072; Skyscan, Aartselaar, Belgium). The scanning protocol was set at an isometric resolution at 8.3 mm, and X-ray energy settings of 80 kV and 80 mA. After reconstruction, BV/TV, trabecular number (Tb.N), trabecular separation (Tb.Sp), and trabecular thickness (Tb.Th) of each sample were measured.

### Hard tissue slicing and Fluorescence microscopy

The specimens were dehydrated in increasing concentrations of alcohol from 70% (v/v) to 100% (v/v) for one week, followed by defatting in xylol for 24 h, then infiltrated and embedded in polymethylmethacrylate (PMMA). Thereafter, sagittal sections with a thickness of approximately 75 μm were cut by microtome (Leitz, Wetzlar, Germany) and investigated with broad-band fluorescence microscopy. Visualization of tetracycline, calcein green, and alizarin red was achieved with Filter 09 (Carl Zeiss MicroImaging GmbH, Jena, Germany). Filter 02 (Carl Zeiss MicroImaging GmbH) was used to investigate alizarin red and calcein blue bands of new bone formation^29^. Fluorescence images were acquired, and the microsections were immersed in xylene until loosening of the cover slips. Collagen content was then examined using Verhoeff-Van Gieson staining (VVG; Elastic Stain Kit; Sigma–Aldrich) according to the manufacturer’s instructions. VVG staining was performed in duplicate to rule out procedural or sample-specific factors that could affect the results^30^.

### Statistical analysis

SPSS 17.0 software package was used for statistical analysis and all values are presented as the mean ± standard deviation (SD). One-way analysis of variance with Bonferroni correction was performed to evaluate statistically significant differences between the three groups. *P* < 0.05 was considered statistically significant.
